# Inositol 1,4,5-trisphosphate receptor type 2 is associated with the bone–vessel axis in chronic kidney disease–mineral bone disorder

**DOI:** 10.1080/0886022X.2022.2162419

**Published:** 2023-01-16

**Authors:** Qiong Xiao, Yun Tang, Haojun Luo, Sipei Chen, Qiao Tang, Rong Chen, Lin Xiong, Jun Xiao, Daqing Hong, Li Wang, Guisen Li, Yi Li

**Affiliations:** a Department of Nephrology, Sichuan Academy of Medical Science and Sichuan Provincial People’s Hospital, School of Medicine, University of Electronic Science and Technology of China, Chengdu, China; b Sichuan Clinical Research Center for Kidney Diseases, Clinical immunology Translational Medicine Key Laboratory of Sichuan Province, School of Medicine, University of Electronic Science and Technology of China, Chengdu, Sichuan, China; c Department of Nephrology, Chinese Academy of Sciences Sichuan Translational Medicine Research Hospital, Chengdu, Sichuan, China; d Department of Stomatology, The First Affiliated Hospital of Chongqing Medical and Pharmaceutical College, Chongqing, China; e Department of Palliative Medicine, Chongqing University Cancer Hospital, Chongqing, China; f Department of Cardiovascular Medicine, Chongqing University Center Hospital, Chongqing, China

**Keywords:** Bone–vessel axis, chronic kidney disease–mineral bone disorder, clinical relevance, inositol 1,4,5-trisphosphate receptor type 2, vascular calcification

## Abstract

**Objective:** The pathogenesis of renal osteopathy and cardiovascular disease suggests the disordered bone–vessel axis in chronic kidney disease–mineral bone disorder (CKD–MBD). However, the mechanism of the bone–vessel axis in CKD–MBD remains unclear.

**Methods:** We established a CKD–MBD rat model to observe the pathophysiological phenotype of the bone–vessel axis and performed RNA sequencing of aortas to identify novel targets of the bone–vessel axis in CKD–MBD.

**Results:** The microarchitecture of the femoral trabecular bone deteriorated and alveolar bone loss was aggravated in CKD–MBD rats. The intact parathyroid hormone and alkaline phosphatase levels increased, 1,25-dihydroxyvitamin D3 levels decreased, and intact fibroblast growth factor-23 levels did not increase in CKD–MBD rats at 16 weeks; other bone metabolic parameters in the serum demonstrated dynamic characteristics. With calcium deposition in the abdominal aortas of CKD–MBD rats, RNA sequencing of the aortas revealed a significant decrease in inositol 1,4,5-trisphosphate receptor type 2 (ITPR2) gene levels in CKD–MBD rats. A similar trend was observed in rat aortic smooth muscle cells. As a secretory protein, ITPR2 serum levels decreased at 4 weeks and slightly increased without statistical differences at 16 weeks in CKD–MBD rats. ITPR2 serum levels were significantly increased in patients with vascular calcification, negatively correlated with blood urea nitrogen levels, and positively correlated with serum tartrate-resistant acid phosphatase 5b levels.

**Conclusion:** These findings provide preliminary insights into the role of ITPR2 in the bone–vessel axis in CKD–MBD. Thus, ITPR2 may be a potential target of the bone–vessel axis in CKD–MBD.

## Introduction

Chronic kidney disease (CKD) is a global public health problem that is receiving increasing attention. The Global Burden of Disease, Injuries, and Risk Factors Study provides comprehensive estimates of CKD burden [1]. The global prevalence of CKD was 9.1% and 1.2 million people died among 697.5 million cases of CKD in 2017. An estimated 119.5 million Chinese individuals have some stage of CKD, with a high overall prevalence of 10.8% [2]. Because of various complications, the mechanism of CKD is extremely complicated.

Chronic kidney disease–mineral bone disorder (CKD–MBD) is a serious complication of CKD that commonly results in the disablement and death of patients [3]. CKD–MBD commonly manifests as mineral bone disorder, vascular calcification, and other ectopic calcifications in patients with CKD, with increased risks of cardiovascular events and death [4–6]. Disruption of normal mineral homeostasis is a major characteristic of CKD–MBD [7]. One or more of the following pathological manifestations may occur: abnormal metabolism of calcium, phosphorus, parathyroid hormone (PTH), or vitamin D; abnormal bone transformation, mineralization, bone mass, linear growth, or strength [8–11]; and calcification of blood vessels or other soft tissues [12]. Previous studies on the pathogenesis of renal osteopathy and cardiovascular disease in CKD have suggested a possible common pathophysiological mechanism in the bone–vessel axis in CKD–MBD [13,14]. However, the molecular mechanisms underlying the regulation of the bone–vessel axis in CKD–MBD are poorly understood. Hence, this study established a CKD–MBD rat model to observe the pathophysiological phenotype and potential regulatory targets involving the bone–vessel axis in CKD–MBD. We hypothesized that some candidate targets may be involved in the bone–vessel axis in CKD–MBD. Further investigations of vascular smooth muscle cells (VSMCs) and patients undergoing maintenance hemodialysis were performed. The current study aimed to explore the regulatory targets of CKD–MBD through systematic research on mechanisms involving the bone–vessel axis.

## Materials and methods

### Study design and participants

We evaluated 73 patients undergoing maintenance hemodialysis (age ≥ 18 years and vintage ≥ 3 months) at the Department of Nephrology of Sichuan Provincial People’s Hospital from January 2018 to December 2020. The criteria for calcification evaluation were scores ≥ 30 Agatston units [15]. The exclusion criteria were as follows: an unwillingness to participate in the research or other intervention studies; complication associated with severe systemic or wasting diseases such as cirrhosis, severe malnutrition, and tumors; hospitalization due to acute disease during the study; undergoing peritoneal dialysis; and use of corticosteroids or immunosuppressants for the past 6 months. Eighteen healthy participants underwent routine health checkups at Sichuan Provincial People’s Hospital during the same period. They were enrolled in the control group. The recorded clinical characteristics included the age and sex of all the participants and the vintage and comorbidities of patients undergoing hemodialysis. This study was approved by the Sichuan Academy of Medical Sciences and Sichuan Provincial People’s Hospital Medical Ethics Committee (no. 2017.36) and conducted in accordance with the principles of the Helsinki Declaration. Written informed consent was obtained before the commencement of the study.

### 5/6 Nephrectomy rat model

The animal treatment protocol was approved by the Institutional Animal Care and Use Committee of the Sichuan Academy of Medical Sciences, Sichuan Provincial People’s Hospital, School of Medicine, University of Electronic Science and Technology of China. All the experiments were conducted in accordance with the National Institutes of Health Guide for the Care and Use of Laboratory Animals. Male Wistar rats (DOSSY, China) aged eight weeks were housed under the standard conditions of a 12-h light/dark cycle, a temperature of 20–26 °C, and a humidity level of 40–70%. Food and water were supplied as required.

**Figure 1.**
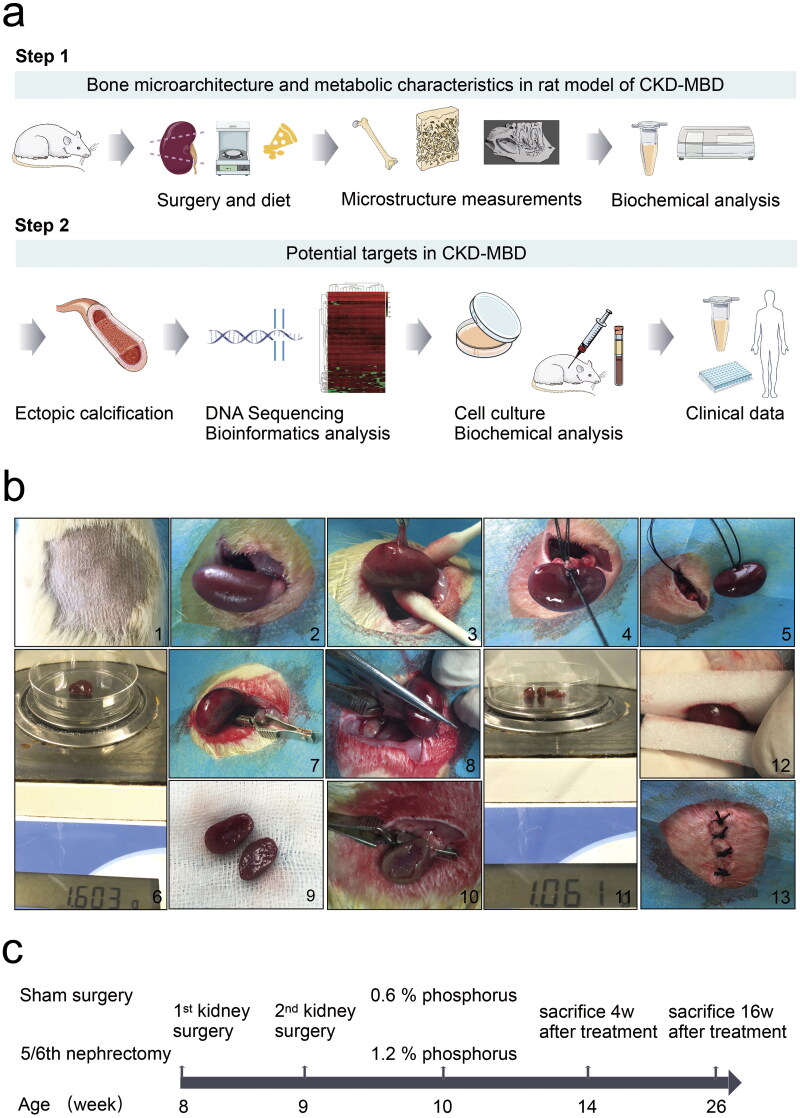
Illustration of the study workflow. a, Schematic overview of potential targets in chronic kidney disease–mineral bone disorder (CKD–MBD) progression. b, A two-step nephrectomy was performed with left-sided uninephrectomy from 1 to 6 and right-sided subtotal nephrectomy from 7 to 13. c, Sham surgery in the sham group, 5/6 nephrectomy in the CKD group. The experimental time points were selected at 4 and 16 weeks, respectively, after surgery with phosphorus diet to mimic the clinical disease status. *N* = 5 for each group.

As presented in Figure 1(b), step-one surgery was a left-sided uninephrectomy, and step-two surgery was a right-sided subtotal nephrectomy. The two surgeries were performed one week apart. Rats were anesthetized by intraperitoneal injection of 1% pentobarbital sodium (4 mL/kg) with prescribed pre- and perioperative analgesia using an ibuprofen oral suspension (0.25 mL/kg, 1:10 dilution in saline solution). The skin in the epidermal projection of the kidney was disinfected with 1% povidone–iodine solution and 75% alcohol. Under anesthesia, a lubricant eye ointment was used to prevent dry eyes. A 1.0-cm incision was made in the skin of the epidermal projection of the kidney parallel to the ribs, using anatomical forceps and blunt scissors. The left kidney was exposed by blunt dissection of the muscles and fascia into the abdominal cavity. Care was taken to separate the renal capsule, remove the adrenal gland, and ligate renal arteries, veins, and ureters. Adrenal glands were not disturbed during the procedure. The renal vessels adjacent to the kidney were cut and the kidneys were removed to avoid cutting off the ends of the ligature. The excised kidneys were dried with gauze for weighing and recording. The incision was closed using a layered counterpoint suture with 4-0 suture for the skeletal muscle and 3-0 suture for the skin. Seven days later, approximately two-thirds of the left kidney’s weight was calculated preoperatively. The right kidney was gently exposed as previously described for the left kidney. A microscopic hemostatic clip was placed at the renal hilum for ≤ 1 min. Sharp scissors were used to resect the upper and lower poles of the right kidney. The excised kidney tissues were weighed to obtain the previous calculation results. If the amount of kidney tissue was insufficient, it was quickly resected. Mild pressure was applied using two pieces of an absorbable gelatin sponge (XIANG’EN, China), sterile gauze was used to cover the residual kidney, and microscopic hemostatic clips were promptly removed. The incision was closed as previously described. The sham group underwent the same procedure, including exposure of the kidney, separation of the renal capsule, retention of the adrenal gland, clipping of the right renal hilum for 1 min, and closure of the wound without extirpation of the left kidney or cutting of the right kidney pole.

The rats were placed on a warming pad that was heated to 40 °C. Rats were observed until they regained consciousness. Subsequently, they were placed in clean solitary cages and injected with 0.05 mL (500 mg/kg) cefazoline sodium intraperitoneally once daily for three days to prevent infection. During follow-up, the 5/6 nephrectomy rats in the CKD group were fed a 1.2% phosphorus diet (XIETONG SHENGWU, China) [16–18]. The rats in the sham group were fed a standard rodent 0.6% phosphorus diet (XIETONG SHENGWU, China) and used as controls. Other components of the diet, including protein, fat, fiber, and total calcium were similar in the CKD and sham groups. The observation period was from 4 to 16 weeks in the CKD and sham groups after the completion of surgery and phosphorus diet to mimic clinical disease status, as summarized in Figure 1(c). Seven rats from each group were subjected to the surgery (*n* = 7).

During the monitoring period, the rats were euthanized if they showed any signs of untreatable infection, walking difficulty, inability to eat or drink, coma, or loss of ≥ 20% of their preoperative weight. All animal experiments were performed in accordance with the ethical standards of the Center for Animal Experiments of the Sichuan Academy of Medical Sciences and Sichuan Provincial People’s Hospital (no. 2017. 36).

### Cell culture

Rat aortic smooth muscle cells (RASMCs) were obtained from the National Collection of Authenticated Cell Culture. Cells were cultured in Dulbecco’s modified Eagle’s medium (C11995500BT, Gibco, China) supplemented with 10% fetal bovine serum (10100147, Gibco, Australia), 100 U/mL penicillin, and 100 U/mL streptomycin (SV30010, HyClone, China) in an atmosphere of 5% CO_2_ at 37 °C. The RASMCs were treated with 10 mM β-glycerophosphate (50020, Sigma-Aldrich, USA) and 1.5 mM CaCl_2_ (C7250, Solarbio, China) for seven days to induce calcification [19,20]. RASMCs treated with medium without β-glycerophosphate or CaCl_2_ were used as controls.

### Specimen preparation

The experimental animals were euthanized to collect femurs and mandibles from the CKD and sham groups. Three left or right femurs and mandibles from five rats in each group were randomly selected for micro-computed tomography (CT) scanning. Femoral bones and mandibles were stored in 4% paraformaldehyde (Biosharp, China) for morphological and structural measurements. The shapes of the bones were trimmed before the experiment using a grinder (90-102, SAESHIN, South Korea). Rat aortas were collected in 10% formalin for alizarin red S staining and quick-frozen in liquid nitrogen for sequencing (FLO-PRO002, Oxford Nanopore Technologies, UK). Rat kidneys were collected for hematoxylin–eosin (HE) and periodic acid–Schiff (PAS) staining.

### HE staining

Rat renal tissues were fixed in 10% formalin, dehydrated, embedded, and cut into 3-µm sections. Following the protocol of the HE staining kit (G1120, Solarbio, China), a hematoxylin solution was used to stain each sample for 5 min, and the samples were then washed for approximately 10 min. Eosin staining solution was added for 1 min, and the sections were washed. Neutral gum was used to seal the sheet, which was photographed under a microscope (3DHISTECH, Pannoramic, Hungary).

### PAS staining

Using a PAS staining kit (G1281, Solarbio, China), 100 μL of periodic acid solution was added to 3-µm sections of the rat renal tissue, reacted in a wet box for 10 min away from light, and washed for 5 min. One hundred microliters of Schiff reagent were added and incubated for 1 h in the dark at 37 °C and then washed for 5 min. Hematoxylin staining was performed for 30 s, and the sections were washed twice, sealed, and photographed (3DHISTECH, Pannoramic, Hungary).

### Alizarin red S staining

Alizarin red S staining (G3280, Solarbio, China) was used to detect calcium deposition. Three-micrometer sections of rat arterial tissues and RASMCs were fixed in 10% formalin and incubated with 2% alizarin red S solution for 15 min, McGee-Russell for 5 s, and hematoxylin for 5 min before washing with deionized distilled water for 5 min. Arterial tissue slices were then sealed with neutral gum and imaged under a microscope. The RASMCs in the six-well plates were photographed using a camera (6 D, Canon, Japan).

### Biochemical analysis

The rats were placed in metabolic cages to collect urine for 24 h, and blood samples were collected from rat hearts after 12 h of fasting before sacrifice. Blood samples were centrifuged for 15 min at 3000 rpm at 4 °C and frozen at −80 °C until use. Urine levels of creatinine (UCr), albumin (Alb), albumin-to-creatinine ratio (ACR), serum creatinine (CRE), and blood urea nitrogen (BUN) were measured using an autoanalyzer system (Cobas 8000, Roche, Germany). The serum levels of intact parathyroid hormone (iPTH) (ZC-54451, ZCI BIO, China), intact fibroblast growth factor-23 (FGF23) (ZC-36459, ZCI BIO, China), 1,25-dihydroxyvitamin D3 (1,25-(OH)2-D3) (ZC-37681, ZCI BIO, China), alkaline phosphatase (ALP) (ZC-36805, ZCI BIO, China), osteocalcin (OCN) (ZC-36660, ZCI BIO, China), procollagen type I N-terminal propeptide (PINP) (ZC-36129, ZCI BIO, China), bone sialoprotein (BSP) (ZC-36667, ZCI BIO, China), C-terminal telopeptide of type I collagen (CTX-I) (ZC-35983, ZCI BIO, China), tartrate-resistant acid phosphatase 5 b (TRACP-5B) (ZC-55533, ZCI BIO, China), and inositol 1,4,5-trisphosphate receptor type 2 (ITPR2) (ZC-55529, ZCI BIO, China) were determined using enzyme-linked immunosorbent assay (ELISA) kits according to the manufacturer’s instructions. ELISA kits were placed at room temperature of 25 °C in advance for 20 min; the rat serum was melted on ice; standard wells were set and 50 µL of each standard of different concentrations was added; serum sample wells of rats in CKD and Sham groups were set, and 50 µL of sample was added to each well, 3 vice wells for each sample, *n* = 3; blank wells were set; 100 µL horseradish peroxidase-labeled detection antibody was added to both standard and sample wells, not to blank wells, and incubated at 37 °C for 60 min; the liquid was discarded in the plate, 350 µL of washing liquid was added, and repeated five times, avoiding light during the whole process; 50 µL of substrate A and substrate B were added to each well, and incubated at 37 °C for 15 min in the dark; 50 µL of stop solution was added to each well, and the optical density of the samples was measured using a microplate reader (Model 680, Bio-Rad, USA) at 450 nm within 5 min. The sample concentrations were calculated according to the standard curve.

Fasting serum samples were collected from the patients before the first hemodialysis session. Serum samples were centrifuged and preserved as described above. Biochemical tests for CRE, BUN, serum calcium (CA), serum phosphorus (P), PTH, alkaline phosphatase (ALP), and intact FGF23 were performed at the central laboratory of Sichuan Provincial People’s Hospital. The levels of human 1,25-(OH)2-D3 (ZC-35899, ZCI BIO, China), OCN (ZC-33162, ZCI BIO, China), PINP (ZC-31964, ZCI BIO, China), BSP (ZC-33179, ZCI BIO, China), CTX-I (ZC-31680, ZCI BIO, China), TRACP-5B (ZC-55540, ZCI BIO, China), and ITPR2 (ZC-55524, ZCI BIO, China) were measured using ELISA kits according to the manufacturer’s instructions.

### Micro-CT scanning parameters and three-dimensional reconstruction

The femurs and mandibles in the CKD and sham groups were scanned using a micro-CT imaging system (VivaCT40, SANCO Medical AG, Switzerland) to quantify the microarchitecture of the region of interest (ROI). The micro-CT V6.1 software was used to reconstruct and analyze the images. A three-dimensional Gaussian filter was used to reduce noise in the images [21]. Femurs and mandibles were fixed in sample cups with parallel long axes for top–down micro-CT scanning. The parameters were an integration time of 250 ms, energy of 70 kVp and 114 μA, and an image matrix of 2048 pixels × 2048 pixels. A 0.5-mm aluminum filter was used to control the beam hardening, and the beam hardening correction was 1200 mg·cm^−3^. An area 1 mm below the growth plate of the femur, where the growth plate had disappeared, was scanned as the starting point. The scanning field continued for 1 mm as the endpoint of the ROI, with a 15-um voxel size and 67 slides [22]. The cortical and cancellous bones of the femur were selected manually by the same operator with a control threshold of 205–1000. After threshold processing, the images were evaluated using the following parameters and a unified approach to ensure consistency in all specimen analyses. Bone volume/total volume (BV/TV), bone surface/bone volume (BS/BV), trabecular thickness (Tb.Th), trabecular number (Tb.N), trabecular separation (Tb.Sp), and connectivity density (Conn.D) were measured in cancellous bone. The cortical bone area (Ct.Ar) and cortical thickness (Ct.Th) were measured in the cortical bone.

In the sagittal view of the mandible, the distance from the cementoenamel junction (CEJ) to the alveolar bone crest (ABC) was measured in the distal part of the mandibular first molar. This process was repeated three times [23,24].

### Sequencing and functional enrichment

Total RNA was extracted from rat aortas using TRIzol reagent (15596026, Invitrogen Life Technologies, USA) according to the manufacturer’s instructions. RNA samples met the following criteria: total quantity ≥ 3 μg, concentration ≥ 40 ng/μL and RNA integrity number values ≥ 8.0. Poly (A) mRNA was extracted from the total RNA for purification using mRNA capture beads (VAHTS, N401, China). The sequencing chip was a PromethION flow cell (FLO-PRO002, Oxford Nanopore Technologies, UK) equipped with a flow cell-priming mix (EXP-FLP001 PRO.6, Oxford Nanopore Technologies, UK). Sequencing was performed on a PromethION48 device (PromethION48, Oxford Nanopore Technologies, UK) using MinKNOW software (version 2.2, Nanopore, UK) for 72 h. The Adapter Y top was 5′- GGCGTCTGCTTGGGTGTTTAACCTTTTTTTTTTAATGTACTTCGTTCAGTTACGTATTGCT-3′, and the adapter Y bottom was 5′-GCAATACGTAACTGAACGAAGT-3′. Significantly differentially regulated genes were identified using BMK Cloud (Biomarker Technologies, China) (https://international.biocloud.net) [25] (|Minimum fold change| ≥ 2, *p* < .05) and were annotated using a Gene Ontology (GO) database analysis (http://geneontology.org). Visual analysis was performed using the Wu Kong platform (https://www.omicsolution.com/wkomics/main/) [26] and Xiantao (Xiantao, China) (https://www.xiantao.love) [27].

### Real-time PCR

RASMCs samples were collected, and total RNA was isolated using TRIzol reagent. Real-time quantitative PCR was performed to identify the differential expression of ITPR2 within cells. Reverse transcription of RNA was performed using a PrimeScript RT Reagent Kit (RR047A, TaKaRa, China) on a GeneAmp PCR System (9700, ABI, USA). Real-time PCR was performed using a TB Green Premix Ex Taq II kit (RR820A, TaKaRa, China) on a CFX96 Real-Time PCR Detection System (CFX96 Touch, Bio-Rad, USA). Relative mRNA fold changes were calculated using the 2^–ΔΔCt^ [28] method, with GAPDH as the internal reference. The primers used were as follows: GAPDH-F, GAAGGTCGGTGTGAACGGAT; GAPDH-R, CCCATTTGATGTTAGCGGGAT; ITPR2-F, TGCGGTCTTGGCTCTTATCC; and ITPR2-R, GTCATAGTGGG-AACCCCGTC (TSINGKE Biological Technology, China).

### Statistical analysis

Data are presented as the mean ± standard deviation (SD). Statistical analysis was performed using the GraphPad Prism software (version 8.0, GraphPad Software, USA). The statistical significance of comparisons between the two groups was determined using Student’s unpaired t-test. Data from multiple groups were analyzed using Tukey’s test for multiple comparisons after a one-way analysis of variance. Spearman’s correlation coefficient was used to assess the relationships among vintage, CRE, BUN, PTH, 1,25-(OH)2-D3, OCN, PINP, BSP, CTX-I, TRACP-5B, and ITPR2. Statistical significance was defined as *p* < .05.

## Results

### 5/6 Nephrectomy rat model with high phosphorus facilitated the progression of CKD–MBD in CKD

**Figure 2.**
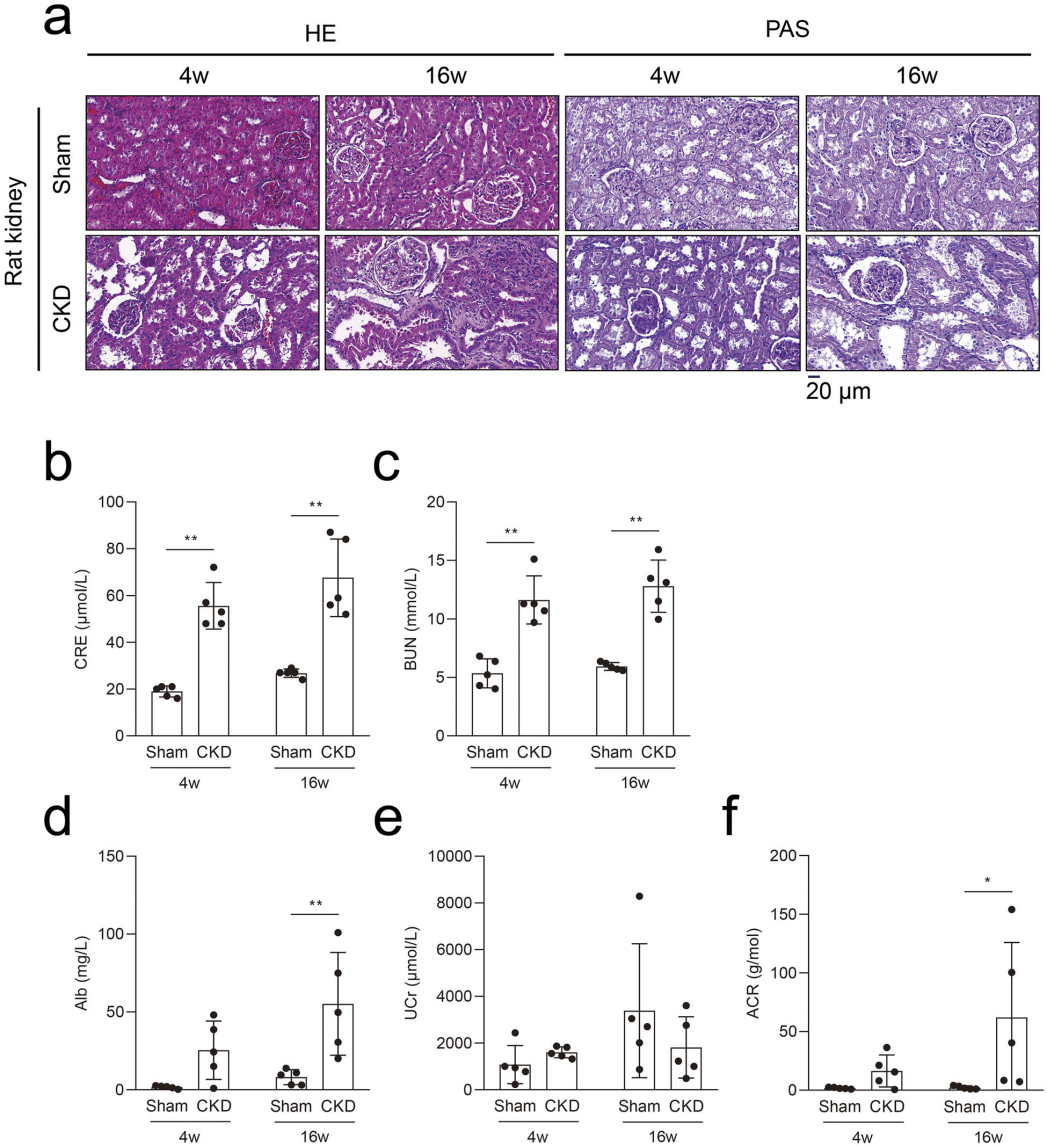
The 5/6 nephrectomy rat model with high phosphorus facilitated the progression of CKD–MBD in CKD. a, Hematoxylin–eosin (HE) staining and periodic acid-Schiff (PAS) staining in rat kidneys in the CKD and sham groups. b and c, Serum biochemical measurements: serum creatinine (CRE) and blood urea nitrogen (BUN) levels in the CKD and sham groups. d–f, Urine biochemical measurements: albuminuria (Alb), urine creatinine (UCr), and albuminuria/urine creatinine (ACR) levels in the CKD and sham groups. *N* = 5 for each group. *p < .05, **p < .01. The scale bar corresponds to 20 μm. The magnification of the microscope was ×400.

A 5/6 nephrectomy rat model induced by high phosphorus levels was established to study potential targets in CKD–MBD (Figure 1(a–c)). Seven rats were euthanized because of an untreatable infection, difficulty walking, or loss of ≥ 20% of the preoperative weight, and one rat was sacrificed unexpectedly (Supplementary Table 1). Twenty rats completed the study, with five rats in each group. The morphological results of the HE and PAS staining showed significant kidney injuries at 4 weeks in the CKD rats, which progressively aggravated at 16 weeks (Figure 2(a)). Compared to the rats in the sham group, significant increases in CRE and BUN levels were observed in CKD rats at 4 and 16 weeks (Figure 2(b,c)). Compared to the rats in the sham group, increased Alb and ACR levels were evident in CKD rats at 16 weeks. No significant difference in the UCr levels was observed between the CKD and sham groups (Figure 2(d–f)).

### Deterioration of the femoral trabecular bone microarchitecture and aggravated alveolar bone loss in the CKD–MBD rats

**Figure 3.**
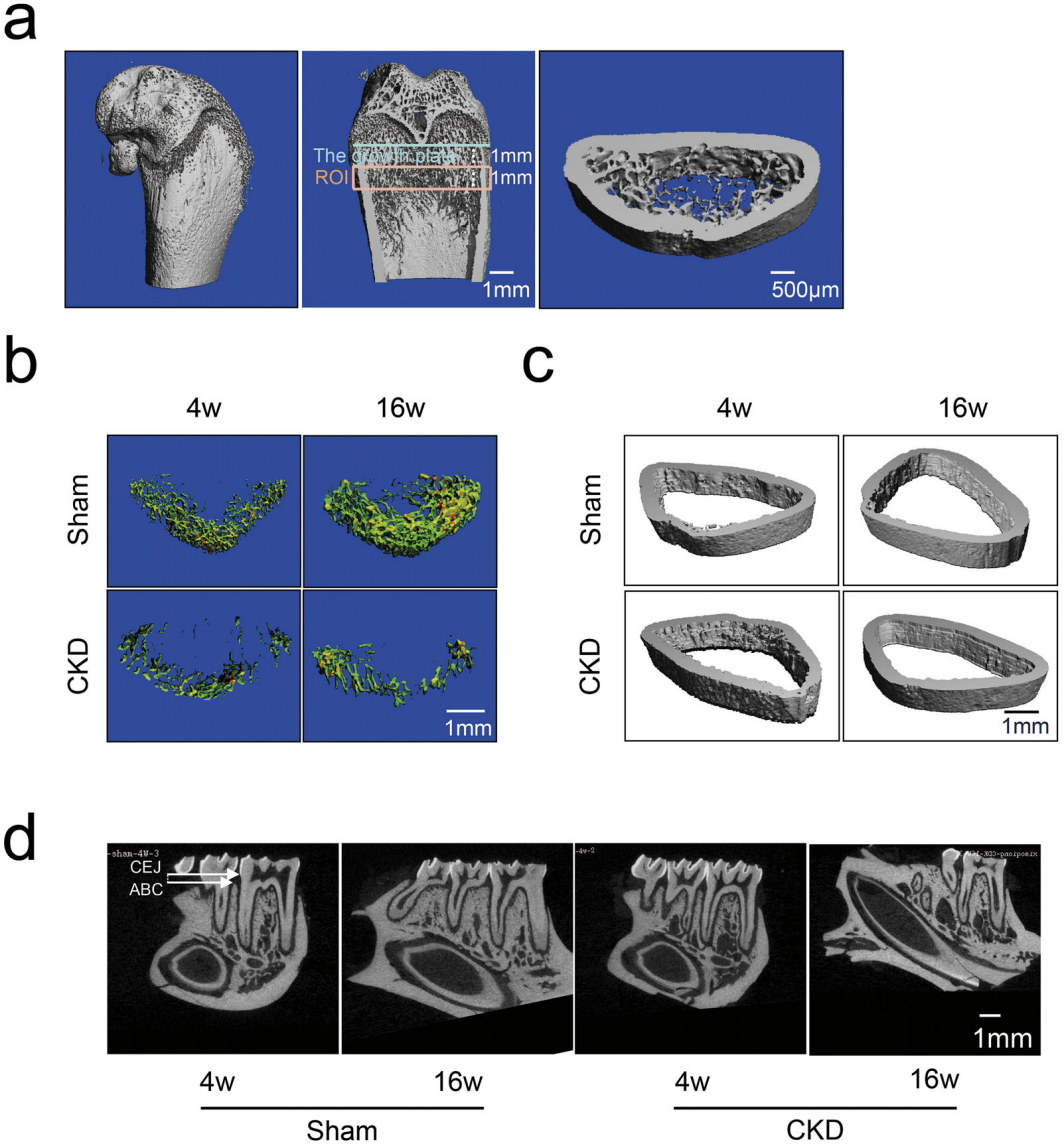
Effects of CKD–MBD on the femoral and alveolar bones of rats by micro-CT scanning in the CKD and sham groups at 4 and 16 weeks. a, The region of interest (ROI) and representative three-dimensional reconstruction images in the femur. An area of 1 mm below the growth plate where the growth plate had disappeared was scanned as the starting point. The scanning field continued for 1 mm as the endpoint of the ROI. b, The representative microarchitecture images of trabecular bone in femur in the CKD and sham groups. c, The representative microarchitecture images of cortical bone in femur in the CKD and sham groups. d, The representative images of alveolar bone on cementoenamel junction (CEJ) to the alveolar bone crest (ABC) distance in the distal of the mandibular first molars in the CKD and sham groups. *N* = 3 for each group. The scale bar ranges from 500 μm to 1 mm.

To determine the effects of CKD–MBD on bone microarchitecture, micro-CT was used to visualize the ROI in the rat femurs (Figure 3(a)). Representative microarchitecture images of trabecular and cortical bones in the femur of the CKD and sham groups are presented (Figure 3(b,c)). The histomorphometry of the trabecular bone and cortical bone in the CKD and sham groups are presented in Table 1. The trabecular bone mass of the CKD group was strikingly reduced compared with that of the sham group, with a decrease in BV/TV at 16 weeks. The CKD group also exhibited deteriorated bone microarchitecture, as shown by the slightly decreased Tb.Th, Tb.N, and Conn.D at 16 weeks. Compared with the sham group, Tb.Sp increased slightly in the CKD group at 16 weeks. The cortical bone microarchitecture did not differ significantly between the CKD and sham groups. These findings suggest that the microarchitecture of the femoral trabecular bone deteriorated in CKD–MBD.

**Table 1.** Bone architectural parameters of femoral and alveolar bones in rats in the CKD and sham groups at 4 and 16 weeks (*n* = 3).

	Sham (4w)	CKD (4w)	Sham (16w)	CKD (16w)
Trabecular bone of femur				
BV/TV (%)	3.133 ± 1.233	3.117 ± 1.948	11.590 ± 2.048	3.250 ± 1.244**
BS/BV (1/mm)	53.853 ± 2.309	46.703 ± 2.985	31.765 ± 0.724	39.891 ± 6.637
Tb.Th (μm)	0.053 ± 0.002	0.061 ± 0.003	0.085 ± 0.002	0.070 ± 0.013
Tb.N (1/mm)	2.341 ± 0.257	2.261 ± 0.175	2.486 ± 0.043	2.211 ± 0.395
Tb.Sp (μm)	0.441 ± 0.033	0.453 ± 0.039	0.400 ± 0.014	0.462 ± 0.072
Conn.D (1/mm^3^)	7.307 ± 3.815	7.049 ± 6.703	23.479 ± 7.449	6.849 ± 5.617*
Cortical bone of femur				
Ct.Ar (mm^2^)	20.927 ± 6.134	16.145 ± 3.990	16.128 ± 2.853	29.050 ± 23.777
Ct.Th (μm)	0.316 ± 0.052	0.371 ± 0.024	0.471 ± 0.043	0.402 ± 0.151
Alveolar bone				
CEJ–ABC (mm)	0.379 ± 0.067	0.448 ± 0.118	0.461 ± 0.073	0.690 ± 0.141**

Abbreviations: BV/TV: bone volume/total volume; BS/BV: bone surface/bone volume; Tb.Th: trabecular thickness; Tb.N: trabecular number; Tb.Sp: trabecular separation; Conn.D: connectivity density; Ct.Ar: cortical bone area; Ct.Th: cortical thickness; CEJ–ABC: cementoenamel junction to the alveolar bone crest distance in the distal of the mandibular first molars. Data are mean ± standard deviation. *p < .05, **p < .01 compared with the sham group at the same time.

Alveolar bone loss was assessed by measuring the distance from the cementoenamel junction to the alveolar bone crest (CEJ–ABC) at the distal site of the mandibular first molars using micro-CT scanning. Normal alveolar bone was observed in the sham group, whereas loss of alveolar bone was observed in the CKD group in the representative images (Figure 3(d)). Quantitative data on alveolar bone showed that the CEJ–ABC distance in the CKD group was increased compared with that in the sham group at 16 weeks (Table 1). A longer CEJ–ABC distance suggested a greater effect on alveolar bone loss. This finding indicates that CKD–MBD significantly aggravated alveolar bone resorption.

### Characteristic metabolic parameters of the serum in the CKD–MBD rats

**Figure 4.**
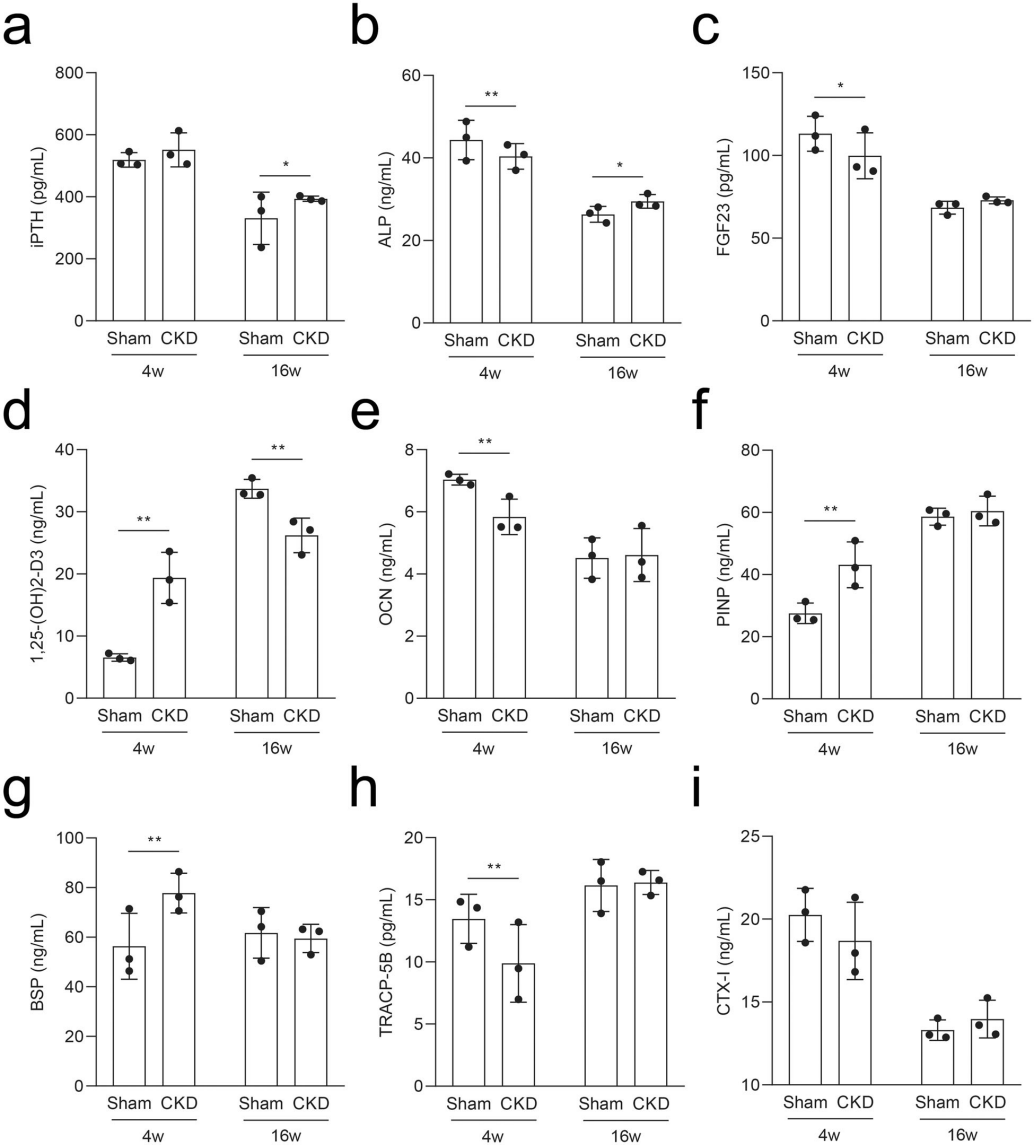
Serum levels of the rats in the CKD and sham groups were analyzed by ELISA at 4 and 16 weeks. a–i, The serum levels of intact parathyroid hormone (iPTH), alkaline phosphatase (ALP), intact fibroblast growth factor 23 (FGF23), 1,25-dihydroxyvitamin D3 (1,25-(OH)2-D3), osteocalcin (OCN), procollagen type I N-terminal propeptide (PINP), bone sialoprotein (BSP), tartrate-resistant acid phosphatase 5b (TRACP-5B), and C-terminal telopeptide of type I collagen (CTX-I). *p < .05, **p < .01, *n* = 3 for each group, each point represents the average value of the three vice wells.

To determine the pathological characteristics of CKD–MBD, we measured the metabolic parameters in rat serum using ELISA. Rats in the CKD group had elevated iPTH and ALP levels at 16 weeks compared to those in the sham group (Figure 4(a,b)). Compared to the sham group, the intact FGF23 levels decreased at 4 weeks and were not significantly different at 16 weeks in the CKD group (Figure 4(c)); serum 1,25-(OH)2-D3 levels increased at 4 weeks and then decreased at 16 weeks in the CKD group (Figure 4(d)). The levels of OCN in the CKD group decreased at 4 weeks, while the levels of PINP increased, and both returned to the mean level at 16 weeks compared to those in the sham group (Figure 4(e,f)). The levels of BSP increased and TRACP-5B decreased in the CKD group at 4 weeks and both returned to the mean level at 16 weeks compared to those in the sham group (Figure 4(g,h)). The CTX-I levels showed no significant difference (Figure 4(i)). Thus, bone resorption and formation are dynamic characteristics throughout CKD–MBD.

### RNA sequencing identified differentially expressed genes in the aortas of CKD–MBD rats

**Figure 5.**
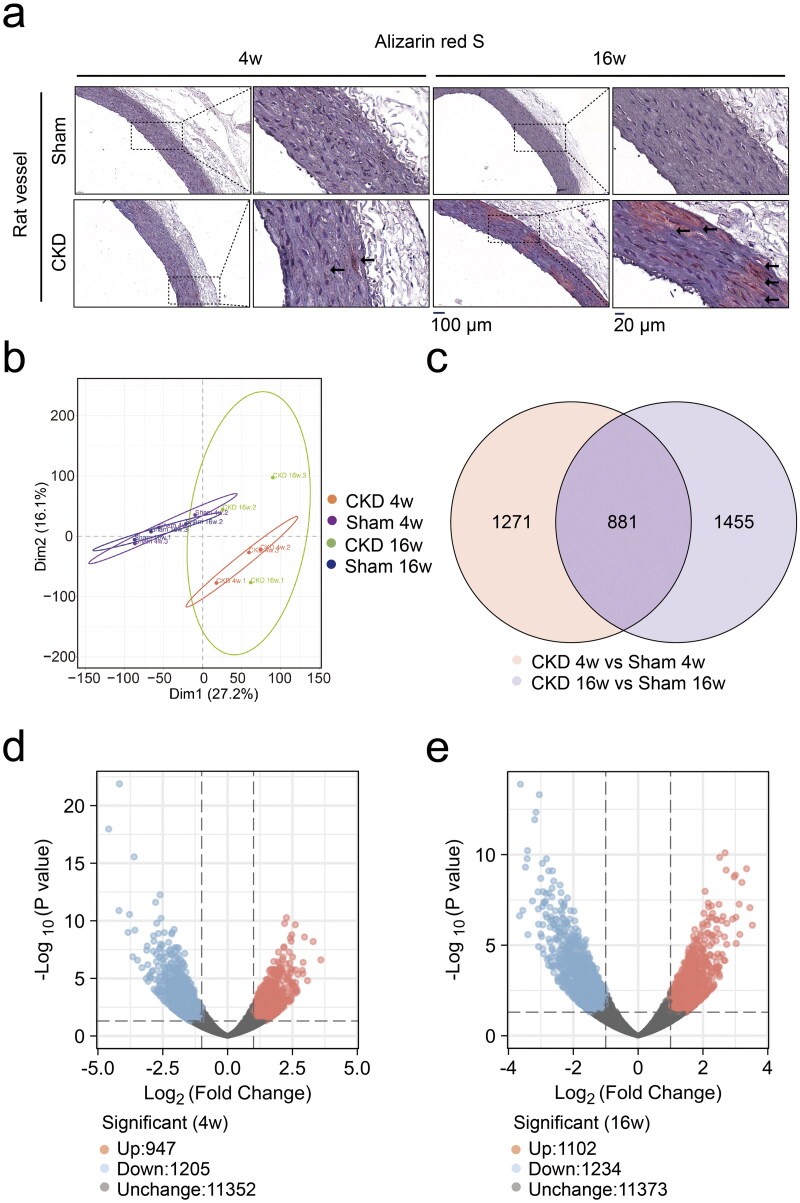
RNA sequencing identified differentially expressed genes in the aortas of CKD–MBD rats at 4 and 16 weeks. a, Alizarin red S staining of rat abdominal aortas. The scale bar ranges from 20 μm to 100 μm. b, Principal component analysis plot of the mRNA expression profiles. c, Venn chart of the numbers of differentially expressed genes. d and e, Volcano plot analysis of differentially expressed genes at 4 and 16 weeks, |Log_2_FC| > 1 and *p* < .05. *N* = 3 for each group.

Alizarin red S staining demonstrated aggravated calcium deposition in rat abdominal aortas in the CKD group from 4 to 16 weeks (Figure 5(a) and Supplementary Figure 1). Full-length RNA sequencing analysis of rat aortas was performed to identify differentially expressed genes. Principal component analysis of mRNA expression showed differential expression profiles between the CKD and sham groups (Figure 5(b)). The Venn chart displayed 2152 and 2336 differentially expressed genes at 4 and 16 weeks, respectively, with an intersection of 881 genes (Figure 5(c)). The volcano plot showed that compared to those in the sham group, 947 genes were upregulated and 1205 genes were downregulated at 4 weeks (Figure 5(d)), whereas 1102 genes were upregulated and 1234 genes were downregulated at 16 weeks in the CKD group (|Log_2_FC| > 1 and *p* < .05) (Figure 5(e)).

### RNA sequencing identified that ITPR2 was significantly downregulated in the aortas of the CKD–MBD rats

**Figure 6.**
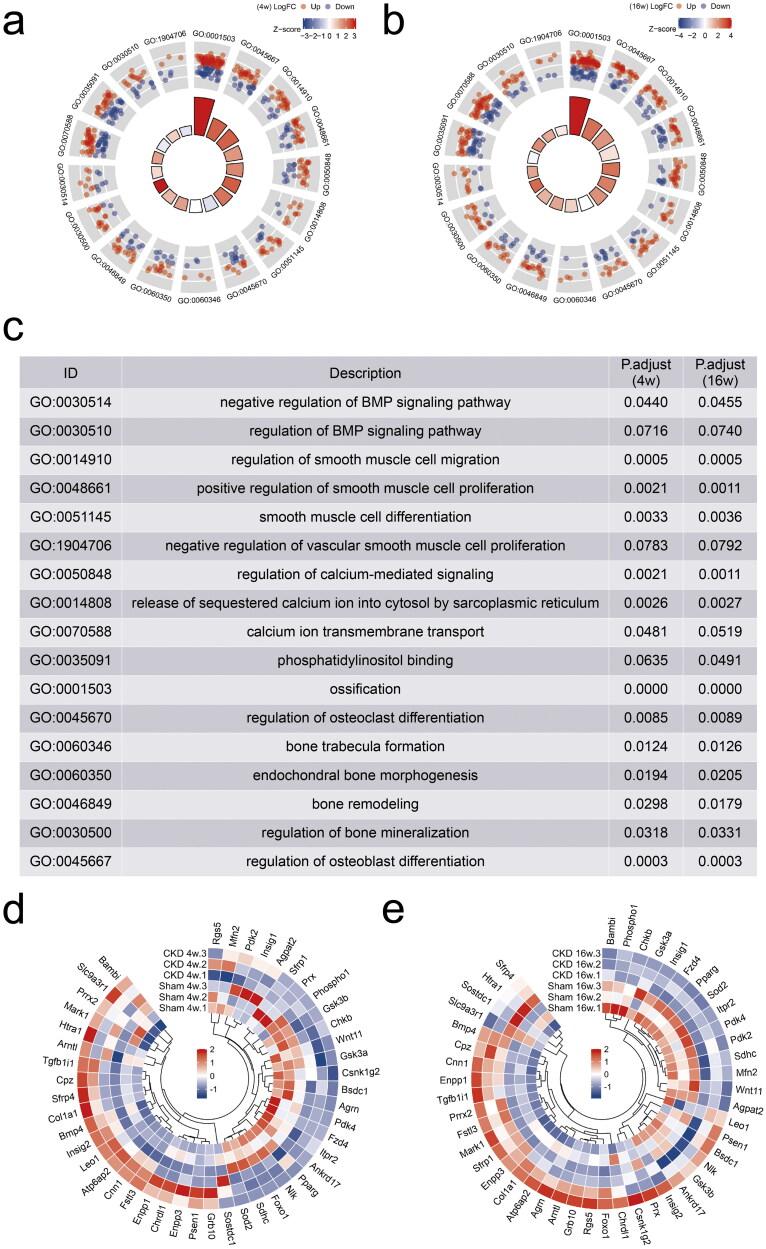
RNA sequencing identified inositol 1,4,5-trisphosphate receptor type 2 (ITPR2) significantly downregulated in the aortas of CKD–MBD rats. a and b, Gene Ontology (GO) databases analysis representatively on the differentially expressed genes at 4 and 16 weeks. c, Description of GO databases analysis representatively. d and e, Clustering heatmap of 46 differentially expressed genes between the CKD and sham groups at 4 and 16 weeks. The experiment was conducted in triplicates, |Fold change| ≥ 2, *p* < .05.

The differentially expressed genes were representatively annotated in the BMP signaling pathway, smooth muscle cell differentiation, calcium ion transmembrane transport, and bone morphogenesis/remodeling/mineralization using the GO database (Figure 6(a–c)). The characterization of the 46 differentially expressed genes was performed using hierarchical cluster analysis at 4 and 16 weeks (Figure 6(d,e)). The Log_2_FC of ITPR2 at 4 and 16 weeks was −1.3693 (*p* = .0047) and −2.3907 (*p* = .000), respectively (Supplementary Table 2). ITPR2 was significantly downregulated in the CKD group at 4 and 16 weeks.

### ITPR2 was downregulated in RASMCs after high-phosphorus treatment, and was first downregulated and then slightly upregulated in the serum of the CKD–MBD rats

**Figure 7.**
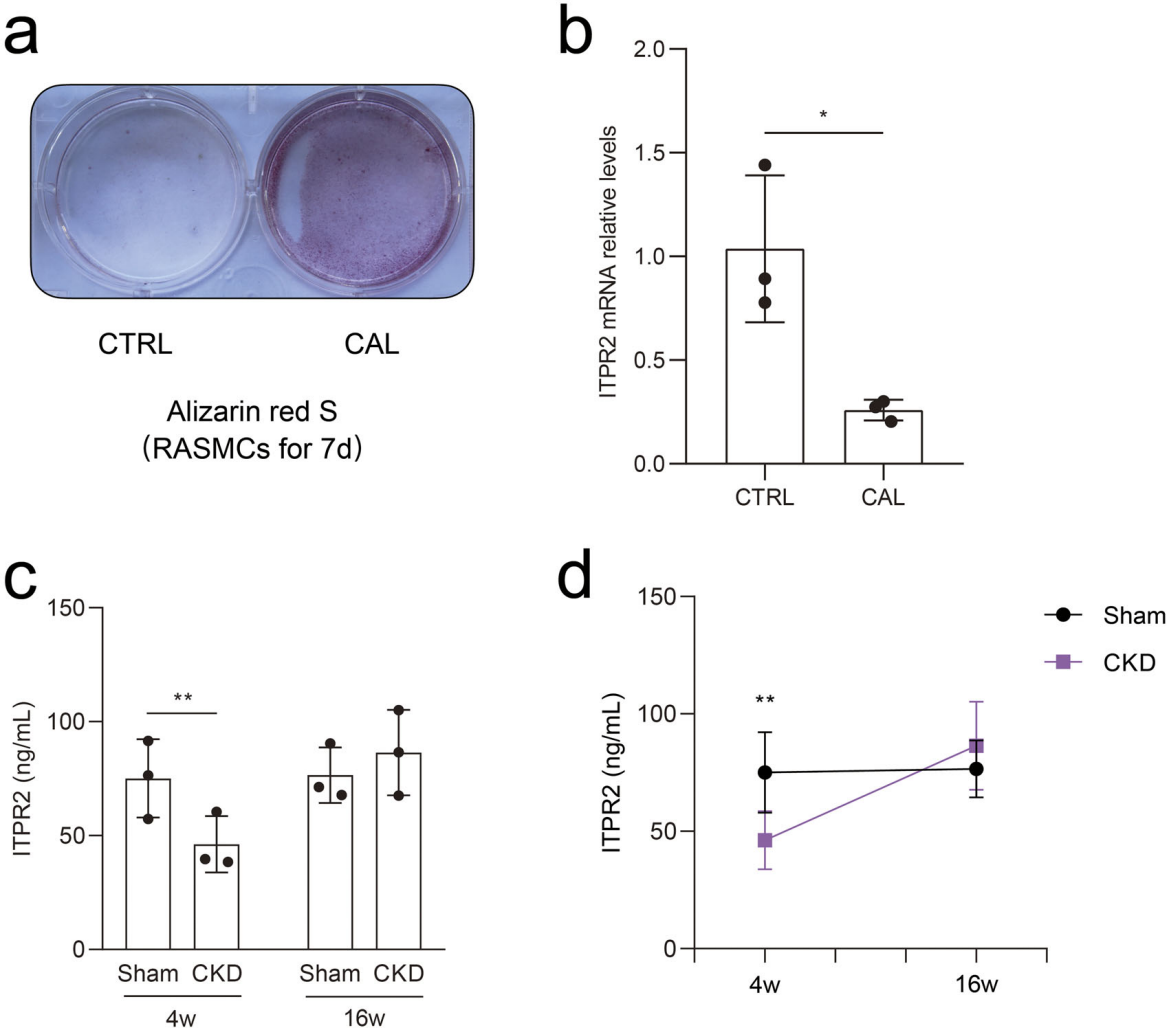
Differential expression of ITPR2 *in vitro* and *in vivo*. a, Alizarin red S staining detected calcium deposition in rat aortic smooth muscle cells (RASMCs) with and without high-phosphorus medium for seven days, denoted as the calcification group (CAL) and the non-calcification group (CTRL), respectively. b, Expression levels of ITPR2 in RASMCs, as assayed by real-time PCR. *N* = 3 for each group. c, Serum levels of ITPR2 were determined by ELISA in rats in the CKD and sham groups. Each point represents the average value of the three vice wells. d, Point-fold line chart in serum ITPR2 levels by ELISA in rats in the CKD and sham groups. *p < .05, **p < .01, *n* = 3 for each group.

Alizarin red S staining revealed significant calcium deposition in RASMCs treated with β-glycerophosphate and CaCl_2_ for seven days (Figure 7(a)). Real-time PCR results showed that the mRNA expressions of ITPR2 significantly decreased in RASMCs after high-phosphorus treatment compared to that in the normal control group (Figure 7(b)). ELISA assessment of the serum ITPR2 levels revealed a significantly decrease in rats in the CKD group at 4 weeks and a slightly increasing trend without statistical difference at 16 weeks compared to rats in the sham group (Figure 7(c)). A point-fold line chart showed that the serum ITPR2 levels were similar in rats in the sham group between 4 and 16 weeks but had an upward trend in the CKD group from 4 weeks to 16 weeks (Figure 7(d)).

### Significant correlation between ITPR2 and TRACP-5B in the vascular calcification group of patients undergoing maintenance hemodialysis

**Figure 8.**
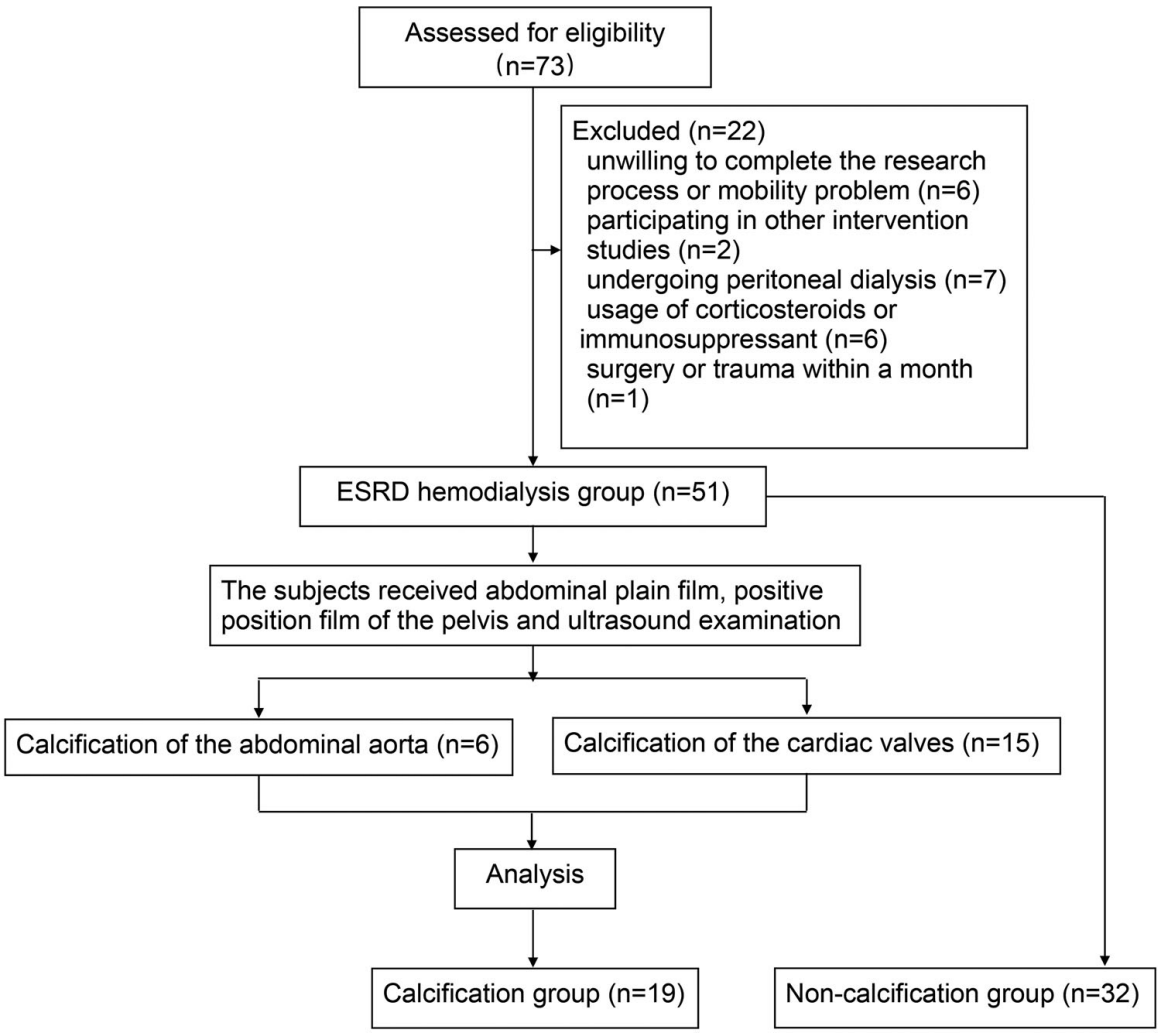
Flowchart of the trial describing the participant selection process.

**Figure 9.**
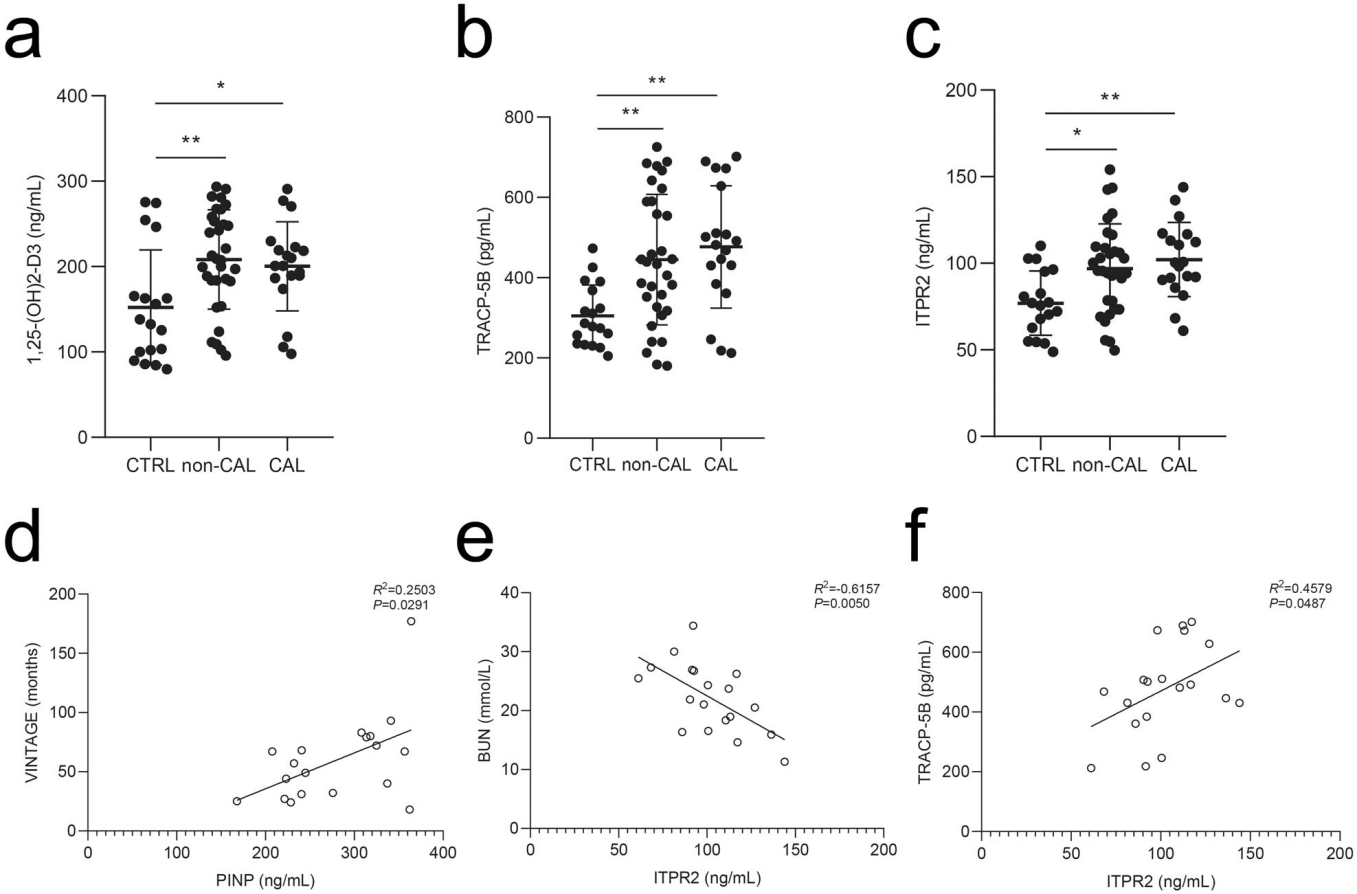
Serum levels of participants were analyzed by ELISA and correlation analyses of the patients undergoing maintenance hemodialysis with vascular calcification were performed. a–c, Serum 1,25-(OH)2-D3, TRACP-5B, and ITPR2 levels in patients undergoing maintenance hemodialysis with vascular calcification (CAL), without vascular calcification (non-CAL), and healthy participants (CTRL). d, Correlation analysis between VINTAGE and PINP. e, Correlation analysis between BUN and ITPR2. f, Correlation analysis between TRACP-5B and ITPR2. *p < .05, **p < .01.

From January 2018 to December 2020, 73 potentially eligible patients were enrolled and 22 were excluded for different reasons (Figure 8). Finally, 51 (69.9%) eligible patients undergoing hemodialysis were included in the study (19 and 32 participants in the calcification and non-calcification groups, respectively). Eighteen healthy individuals were included as negative controls. Table 2 lists the baseline characteristics of the participants, including age, male sex, vintage, comorbidities, and CRE, BUN, CA, P, PTH, ALP, and intact FGF23 levels. The mean age was 54.2 ± 10.2 (range, 31–76) years, and 25 participants (49.0%) were male in the hemodialysis group. The serum levels of 1,25-(OH)2-D3, TRACP-5B, and ITPR2 remained high levels in patients undergoing hemodialysis compared to those in healthy controls (Figure 9(a–c)). The OCN, PINP, BSP, and CTX-I levels did not differ significantly between the groups (Supplementary Figure 2(a–d)). PINP expression was positively correlated with vintage levels (linear correlation coefficient = 0.2503, *p* < .05) (Figure 9(d)), ITPR2 expression was negatively correlated with BUN levels (linear correlation coefficient = −0.6157, *p* < .05) (Figure 9(e)), and positively correlated with TRACP-5B levels (linear correlation coefficient = 0.4579, *p* < .05) (Figure 9(f)) in the vascular calcification group of patients undergoing hemodialysis. No significant correlations were observed between other bone metabolic parameters (Supplementary Figures 3–5).

**Table 2.** Baseline characteristics of the participants.

	Hemodialysis group		Control group
	CAL (*n* = 19)	Non-CAL (*n* = 32)	(*n* = 18)
Age (yeas)	56.6 ± 10.5	52.7 ± 9.9	59.7 ± 17.8
Male	11 (57.9%)	14 (43.8%)	8 (40%)
Vintage (months)	57 (18–177)	41 (12–134)	N/A
Comorbidities			
Diabetes	4 (21.1%)	2 (6.3%)	N/A
Hypertension	17 (89.5%)	31 (96.9%)	N/A
Serum tests			
CRE (μmol/L)	840 (390–1838)^++^	976 (392–1570)^++^	68.9 (45.3–83)
BUN (mmol/L)	21.9 (11.3–34.4)^++^	25.3 (5.8–41.3)^++^	5.9 (3.2–7.2)
CA (mmol/L)	2.4 (2.0–2.7)^#^	2.1 (1.4–2.7)^++^	2.5 (2.3–2.5)
P (mmol/L)	1.6 (1–3)	1.8 (0.7–2.9)	N/A
PTH (pg/mL)	288.9 (40.5–3197)	188 (5.4–1050)	N/A
ALP (U/L)	87 (16.9–158)	100 (60–271)	N/A
FGF23 (pg/mL)	184.9 (41.5–437)^#^	79.9 (1.7–443.7)	N/A

*Notes:* Data on age are presented as mean ± standard deviation. Data on male participants and comorbidities are presented as n (%). Data of vintage and serum tests are presented as median (range).

Abbreviations: CAL: calcification; CRE: serum creatinine; BUN: blood urea nitrogen; CA: serum calcium; P: serum phosphorus; PTH: parathyroid hormone; ALP: alkaline phosphatase; FGF23: intact fibroblast growth factor 23; N/A: not available. # *p* < .05, compared to participants of non-CAL, + *p* < .05, ++ *p* < .01, compared to participants in control group.

## Discussion

CKD–MBD is characterized by abnormalities in laboratory indicators, bone lesions, and calcification of vessel and soft tissues [3]. For abnormal laboratory indicators, the increase in PTH and intact FGF23 levels with a reduction in 1,25-(OH)2-D3 levels in patients with CKD could indicate a disorder of renal function, glomerular filtration function, and renal phosphate excretion in CKD–MBD [29–31]. To elucidate the mechanism of CKD–MBD, the current study established a 5/6 nephrectomy rat model with 1.2% phosphorus. We observed significant kidney injury and calcium deposition in the abdominal aortas of rats that underwent 5/6 nephrectomy and received a high-phosphorus diet. Possibly because of the small number of animals and insufficient observation period, the expected elevated intact FGF23 levels were not observed. Although the intact FGF23 levels were not significantly different, the iPTH levels were significantly increased and the 1,25-(OH)2-D3 levels were significantly decreased at 16 weeks in rats that underwent 5/6 nephrectomy with a 1.2% phosphorus diet al.P is not only expressed in osteoblasts and chondrocytes but also in other mineralizing cell types, such as calcified VSMCs, affecting bone mineralization and vascular calcification [32,33]. The ALP levels were significantly increased in the CKD group at 16 weeks. These findings suggest the presence of CKD–MBD in rats with 5/6 nephrectomy and a 1.2% phosphorus diet.

To further study the bone microarchitecture in CKD–MBD, the results of micro-CT scanning in this study revealed significant loss of femoral trabecular bone and alveolar bone in the CKD–MBD rats. Our results are consistent with the findings of Kalaska et al. [34] and Heveran et al. [35], suggesting the destruction of the bone structure in CKD. Consequently, we agree with Miyata et al. [36] and Kitamura et al. [37] that periodontal health needs to be emphasized in patients with CKD to maintain the quality of life and prolong survival. The mechanism of bone disorder underlying CKD–MBD is complex. OCN is an osteoblast-specific secreted protein closely associated with bone formation [38]. BSP contributes to bone resorption by inducing osteoclast genesis and survival and decreasing osteoclast apoptosis [39]. In our study, changes in the serum OCN levels in rats in the CKD group at four weeks demonstrated that bone turnover decreased early in the disease course. The BSP levels in the CKD group increased sharply at four weeks, suggesting a severe degree of bone resorption initiated at the CKD–MBD stage. Moreover, the International Osteoporosis Foundation and the International Federation of Clinical Chemistry recommend the clinical use of serum CTX-I and PINP levels as reference markers for bone resorption and bone formation, respectively [38,40]. The CTX-I levels in this study were not significantly different. The PINP levels were increased and TRACP-5B levels were decreased in rats with CKD–MBD at four weeks compared to those in the sham group. Although OCN, PINP, and CTX-I are easily cleared from kidneys compared to TRACP-5B [41], their clinical association with CKD remains unclear. Therefore, the current study could consider all the above parameters suggesting dynamic characteristics of bone turnover throughout CKD–MBD.

Regarding the unknown mechanisms of CKD–MBD, the results of the RNA sequencing in the present study identified the ITPR2 gene, encoding inositol 1,4,5-trisphosphate receptor 2 protein (IP_3_R2). ITPR2 can regulate calcium fluxes from the endoplasmic reticulum to the mitochondria [42,43] and is crucial for intracellular calcium homeostasis [44]. It can be commonly expressed in osteoclast precursors to regulate intercellular crosstalk between osteoblasts and osteoclasts to stimulate osteoclast differentiation [45]. Furthermore, Li et al. reported that the inositol phosphate metabolism pathway was significantly enriched in the bone of an osteoporosis rat model, suggesting the importance of the inositol phosphate metabolism pathway in bone metabolism diseases [46]. In the current study, RNA sequencing revealed that ITPR2 levels were significantly reduced in the aortas of the CKD–MBD rats. As a secretory protein, the serum ITPR2 levels in the CKD–MBD rats showed a significantly decrease at 4 weeks and a slightly increasing trend without statistical difference at 16 weeks. These levels were significantly increased in the serum of patients undergoing maintenance hemodialysis. Indeed, there is a discrepancy in the serum levels of ITPR2 between the CKD–MBD rat model and patients undergoing maintenance hemodialysis. Although the remnant kidney model is widely used, there are still some differences in the disease status between rat models and humans. Owing to the complicated heredity and environmental factors of patients undergoing maintenance hemodialysis, there might be a discrepancy in the serum levels of ITPR2 between the CKD–MBD rat model and patients. We will further study the precise mechanisms of heredity and environmental factors in patients undergoing maintenance hemodialysis upon ITPR2 secretion. We also agree that the results were influenced by the observation time. It is possible that the patients undergoing maintenance hemodialysis were too advanced in their state, and therefore, upregulation and not downregulation of ITPR2 levels was observed. It is more likely that the observation period was not long enough to observe the upregulation of ITPR2 levels in the rat model. ITPR2 levels were similar in the sham group between 4 and 16 weeks, but showed an upward trend in the CKD group from 4 to 16 weeks. Correlation analysis involving patients undergoing hemodialysis with vascular calcification revealed that serum ITPR2 levels were negatively correlated with BUN levels and positively correlated with TRACP-5B levels. Although we did not distinguish between patients with CKD–MBD and those without CKD–MBD, which would require a further series of systematic evaluation methods, all patients in the calcification group in the correlation analysis have clearly developed CKD–MBD. These reports from Kuroda et al. and Zhang et al. [47,48] support our results that osteoclasts can be activated with an increase in serum ITPR2 levels, leading to the loss of bone mass involving bone disorders. These findings provide preliminary insights into the role of ITPR2 in the potential association between bone and vessels in CKD–MBD.

Vascular calcification is a common complication of CKD involving the bone–vessel axis in CKD–MBD. High phosphorus levels may induce osteogenic transdifferentiation of VSMCs [19,49]. We observed significantly decreased mRNA levels of ITPR2 in rat vascular smooth muscle cells following high-phosphorus treatment, consistent with the sequencing results of the aortas in CKD–MBD rats. This indicated the potential association of ITPR2 with vascular calcification involving the bone–vessel axis in CKD–MBD.

**Figure 10.**
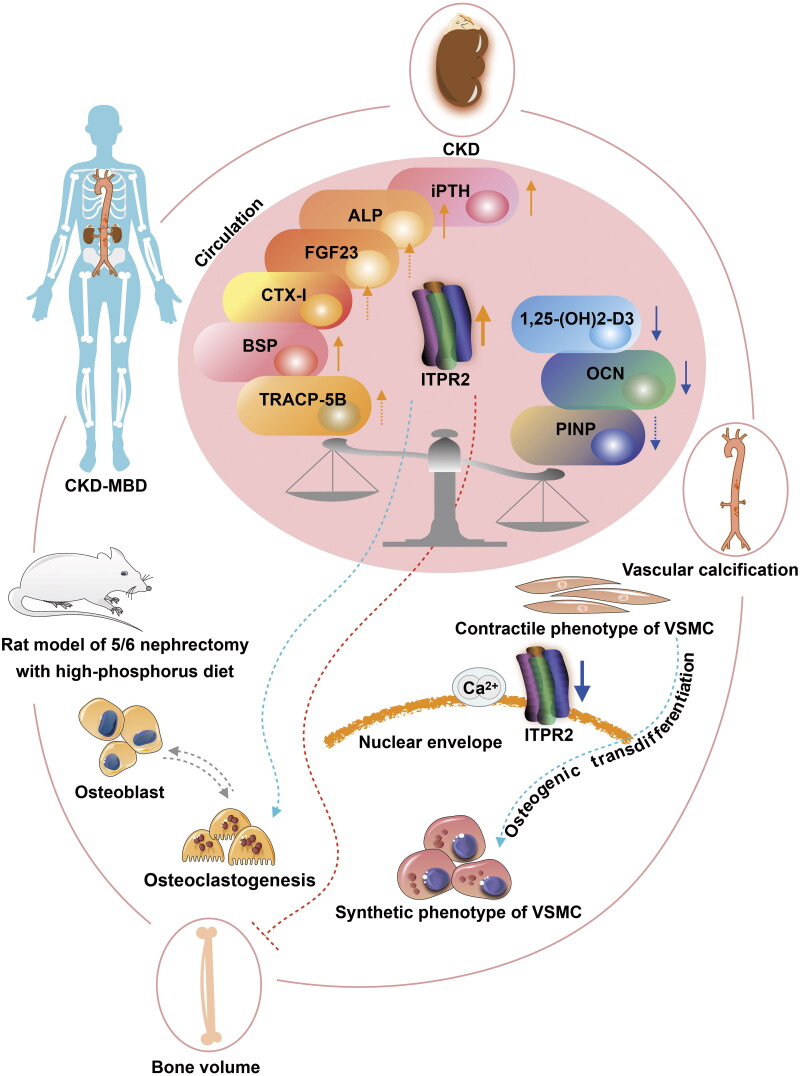
ITPR2 in the potential association of the bone–vessel axis in CKD–MBD. The arrow colors indicate the following: orange, upregulated; blue, downregulated; green, promoted; and red, prevented. Vascular smooth muscle cell (VSMC).

This study had several limitations that warrant discussion. Further evidence and more significant pathological changes can be obtained by increasing the number of experimental animals and extending the observation period. Because of the limitations of the sample size of patients undergoing maintenance hemodialysis, larger cohorts and multicenter studies are needed, including investigating the stratified status of CKD–MBD and the use of medicine. Furthermore, the molecular pathological mechanism of ITPR2 requires exploration in prospective studies, such as the use of phosphate binders, vitamin D preparations, and cinacalcet in basic research, as well as the design of more detailed clinical trials, including the evaluation of medications in CKD–MBD. The current study only observed the preliminary role of ITPR2 associated with the bone–vessel axis in CKD–MBD. Further analysis of the precise role of ITPR2 in the bone–vessel axis in CKD–MBD using transgenic cells and animal models would be interesting. In conclusion, our current study revealed that ITPR2 may be associated with the bone–vessel axis in CKD–MBD (Figure 10). Therefore, ITPR2 may be a potential target of the bone–vessel axis in CKD–MBD.

## Supplementary Material

Supplemental Material

Supplemental Material

Supplemental Material

Supplemental Material

Supplemental Material

Supplemental Material

Supplemental Material

Supplemental Material
