# Supplementary material for: Inositol 1,4,5-trisphosphate receptor type 2 is associated with the bone–vessel axis in chronic kidney disease–mineral bone disorder
Source: Ren Fail. 2023 Jan 16;45(1):2162419. doi: 10.1080/0886022X.2022.2162419 (PMC9848274; doi:10.1080/0886022X.2022.2162419)
Supplement: Supplemental Material [file IRNF_A_2162419_SM7554.pdf]

The method of ELISA

The rats blood samples were collected from hearts after 12 h of fasting before sacrifice. The blood samples were centrifuged for 15 min at 3000 rpm at 4 °C and frozen at −80 °C until use. The serum levels of intact parathyroid hormone (iPTH) (ZC-54451, ZCI BIO, China), serum intact fibroblast growth factor-23 (FGF23) (ZC-36459, ZCI BIO, China), 1,25-dihydroxyvitamin D3 (1,25-(OH)2-D3) (ZC-37681, ZCI BIO, China), alkaline phosphatase (ALP) (ZC-36805, ZCI BIO, China), osteocalcin (OCN) (ZC-36660, ZCI BIO, China), procollagen type I N-terminal propeptide (PINP) (ZC-36129, ZCI BIO, China), bone sialoprotein (BSP) (ZC-36667, ZCI BIO, China), C-terminal telopeptide of type I collagen (CTX-I) (ZC-35983, ZCI BIO, China), tartrate-resistant acid phosphatase 5b (TRACP-5B) (ZC-55533, ZCI BIO, China), FSTL3 (ZC-55528, ZCI BIO, China), and ITPR2 (ZC-55529, ZCI BIO, China) were determined using enzyme-linked immunosorbent assay (ELISA) kits according to the manufacturer’s instructions.

Fasting serum samples were collected from patients before the first hemodialysis session. The serum samples were then centrifuged and preserved as described above. The levels of human 1,25-(OH)2-D3 (ZC-35899, ZCI BIO, China), OCN (ZC-33162, ZCI BIO, China), PINP (ZC-31964, ZCI BIO, China), BSP (ZC-33179, ZCI BIO, China), CTX-I (ZC-31680, ZCI BIO, China), TRACP-5B (ZC-55540, ZCI BIO, China), FSTL3 (ZC-55525, ZCI BIO, China), and ITPR2 (ZC-55524, ZCI BIO, China) were measured using ELISA kits according to the manufacturer's instructions.

Information Classification: General

FGF23 ELISA as an example: 1) FGF23 ELISA kit was placed at room temperature of 25 °C in advance for 20 min; 2) the rat serum was melted on ice and equilibrated slowly to room temperature; 3) standard wells were set and 50 μL of each standard of different concentrations was added; 4) serum sample wells of rats in CKD and Sham groups were set, and 50 μL of sample was added to each well, 3 vice wells for each sample, n = 3; 5) blank wells were set; 6) 100 μL horseradish peroxidase-labeled detection antibody was added to both standard and sample wells, not to blank wells, and incubated at 37 °C for 60 min; 7) the liquid was discarded in the plate, 350 μL of washing liquid was added for 1 minute at room temperature, the plate was dried, and repeated five times, avoiding light during the whole process; 8) 50 μL of substrate A and substrate B were added to each well, and incubated at 37 °C for 15 min in the dark; 9) 50 μL of stop solution was added to each well, and the optical density value of each well was measured at 450 nm using microplate reader within 5 min; 10) the standard curve was made with the R^2 value closed to 1. The sample concentrations were calculated according to the standard curve.

Information Classification: General
