# Supplementary figures and images for: Inositol 1,4,5-trisphosphate receptor type 2 is associated with the bone–vessel axis in chronic kidney disease–mineral bone disorder
Source: Ren Fail. 2023 Jan 16;45(1):2162419. doi: 10.1080/0886022X.2022.2162419 (PMC9848274; doi:10.1080/0886022X.2022.2162419)

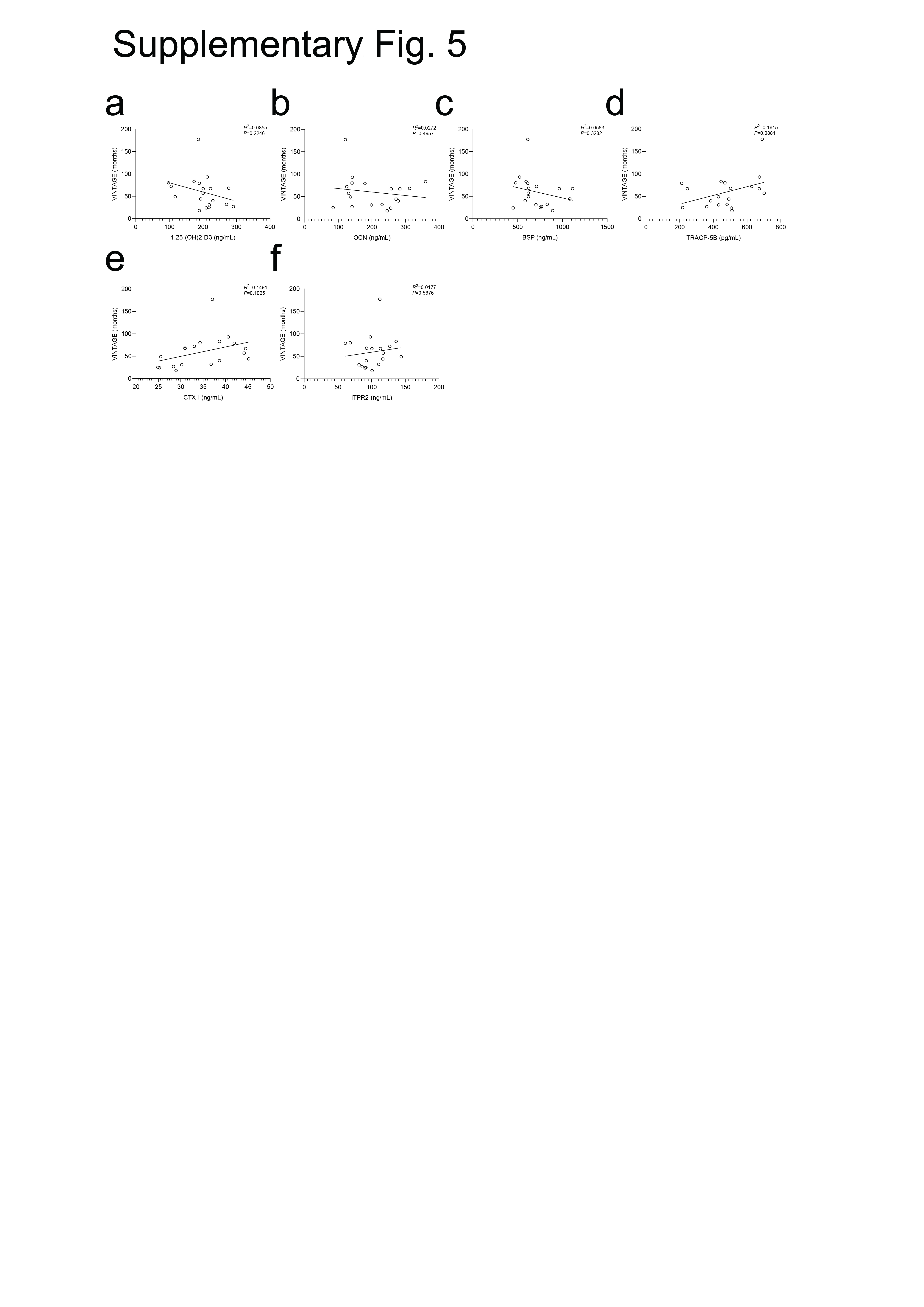

Supplement: Supplemental Material [file IRNF_A_2162419_SM7530.jpg]

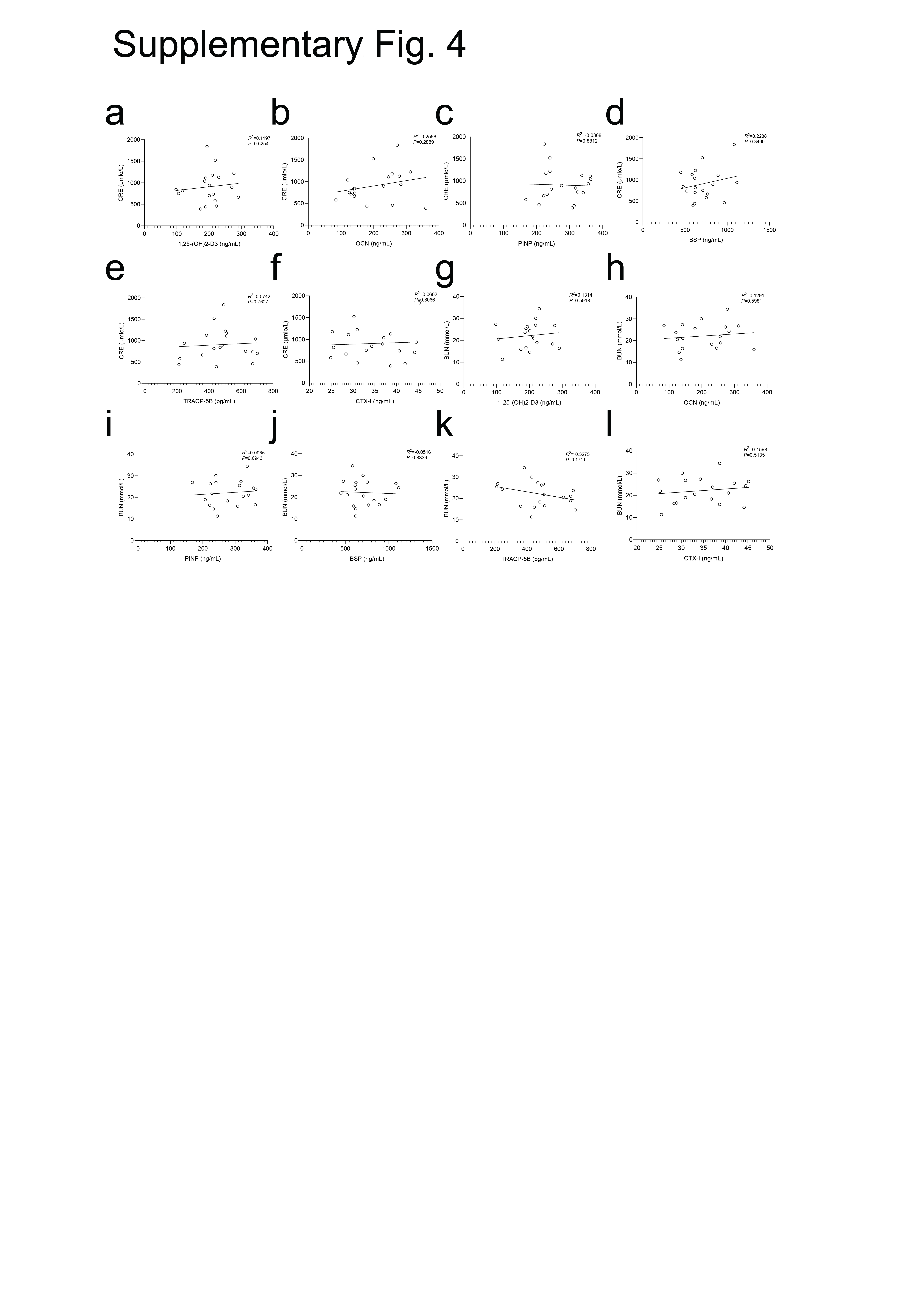

Supplement: Supplemental Material [file IRNF_A_2162419_SM7525.jpg]

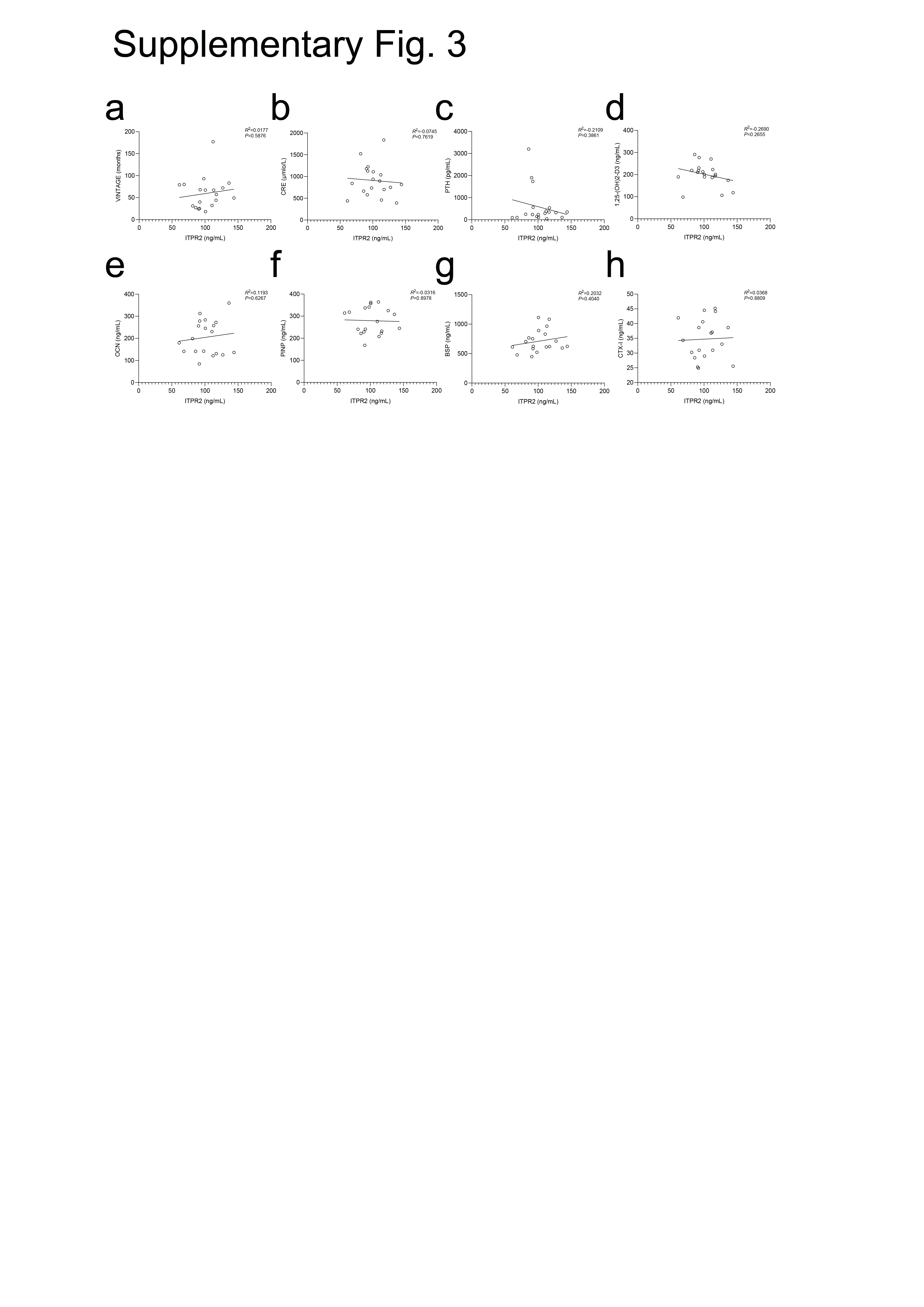

Supplement: Supplemental Material [file IRNF_A_2162419_SM7514.jpg]

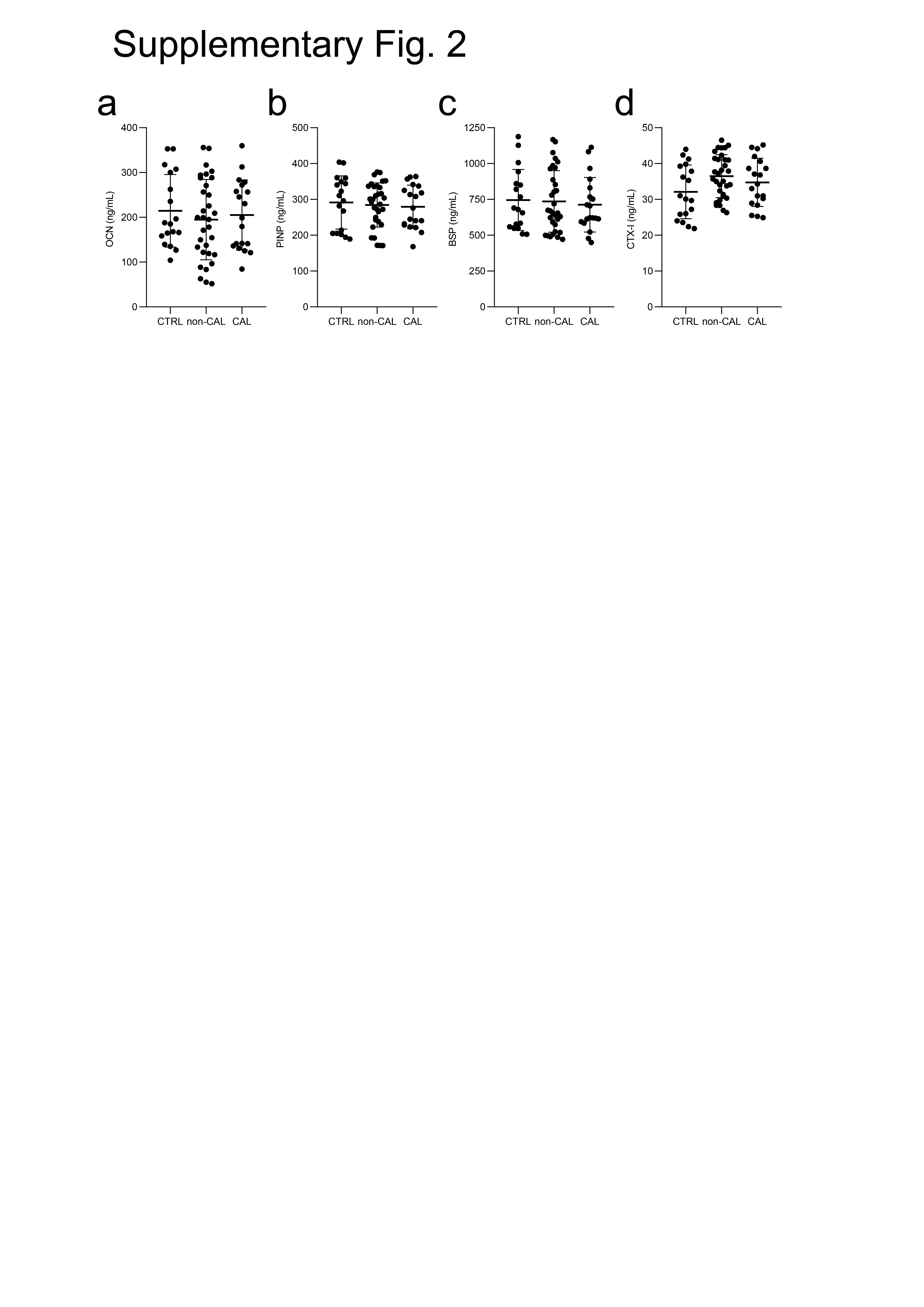

Supplement: Supplemental Material [file IRNF_A_2162419_SM7507.jpg]

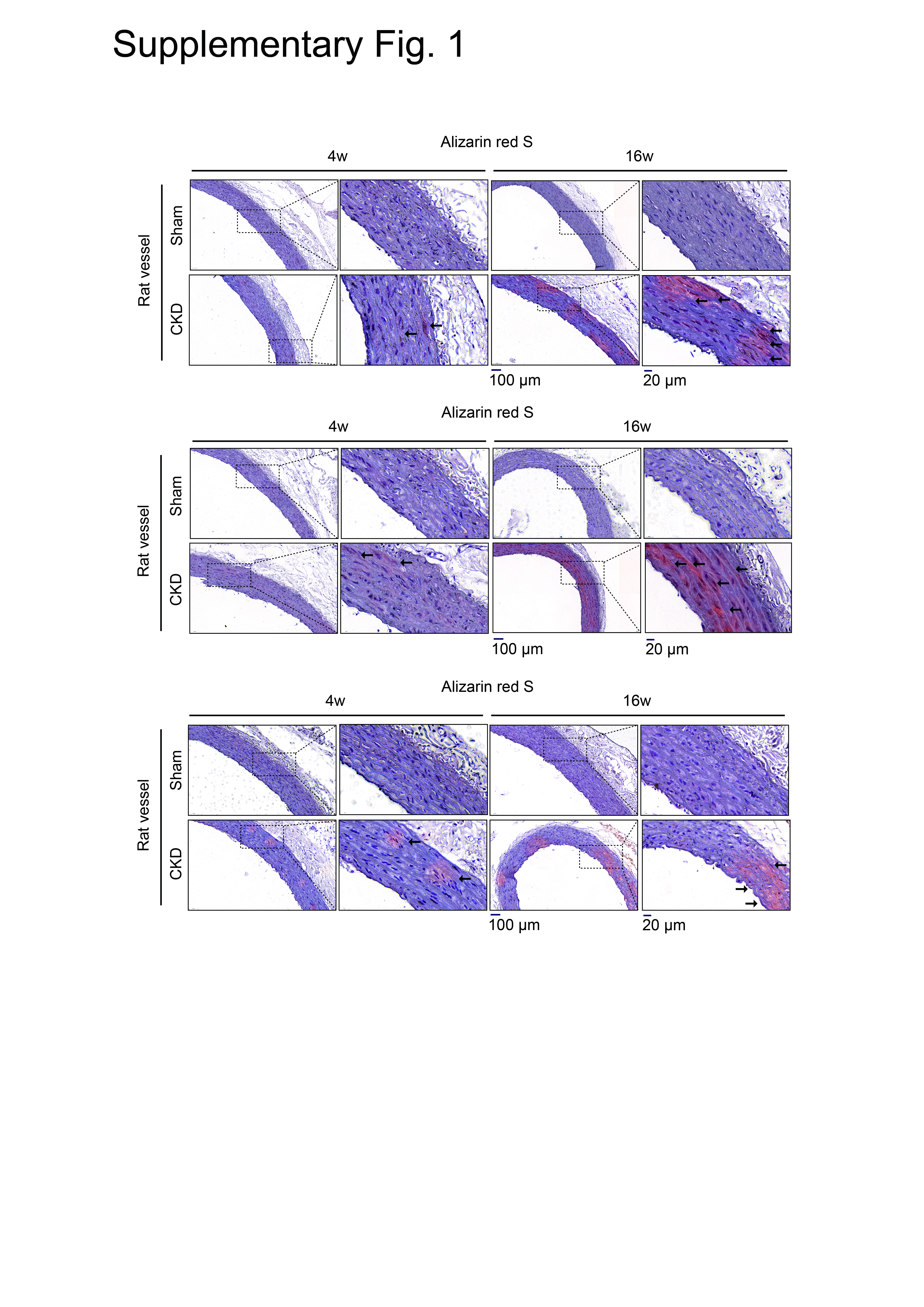

Supplement: Supplemental Material [file IRNF_A_2162419_SM7490.jpg]
